# Supplementary material for: Enhanced Antibacterial and Anti-Inflammatory Activities of the Combination of Cannabis sativa and Propolis Extracts: An In Vitro Study
Source: Int J Mol Sci. 2025 Nov 19;26(22):11181. doi: 10.3390/ijms262211181 (PMC12652737; doi:10.3390/ijms262211181)
Supplement: Supplementary file 1 [file ijms-26-11181-s001.zip › Supplementary File S1 COA CS.pdf]

Center of Plant-produced Biopharmaceutical for Dentistry

ศูนย์พัฒนากระบวนการผลิตชีวเภสัชภัณฑ์จากพืชเพื่อประยุกต์ใช้ทางทันตกรรม จุฬาลงกรณ์มหาวิทยาลัย

คณะทันตแพทยศาสตร์ จุฬาลงกรณ์มหาวิทยาลัย

34 ถ.อังรีดูนัง แขวงปทุมวัน เขตปทุมวัน กรุงเทพฯ

รายงานผลการตรวจวิเคราะห์ทางเคมีของสมุนไพรด้วยเทคนิค HPLC

Report of analysis

**เลขที่รายงาน:**	R65/056
**วันที่รายงาน:**	20 มิถุนายน 2565
**จำนวนหน้าทั้งหมด:**	5
**ชื่อลูกค้า:**	บริษัท ลีพดีแลบ จำกัด
**ที่อยู่:**	89/578 หมู่ 8 บางครุ พระประแดง สมุทรปราการ
**โทร:**	-
**E-mail:**	leapdelab@gmail.com
**วันที่รับตัวอย่าง:**	15 มิถุนายน 2565
**วันที่ทดสอบ:**	15 มิถุนายน 2565
**วิธีทดสอบ:**	In-house method HPLC system
**ห้องปฏิบัติการ:**	ศูนย์พัฒนากระบวนการผลิตชีวเภสัชภัณฑ์จากพืชเพื่อประยุกต์ใช้ทางทันตกรรม จุฬาลงกรณ์มหาวิทยาลัย

ผู้ทดสอบ:

แสดงในหน้าสุดท้าย

ผู้อนุมัติ:

(ลงนาม) .... หัวหน้าฝ่ายวิเคราะห์

(รศ.ภญ.ดร.สรกนก วิมลมั่งคั่ง)

วันที่ 20 มิถุนายน 2565

หมายเหตุ:

1. ห้องปฏิบัติการยืนยันผลการทดสอบเฉพาะต่อตัวอย่างที่นำมาทดสอบเท่านั้น
2. ไม่อนุญาตให้มีการแก้ไข เพิ่มเติม เปลี่ยนแปลงรายงาน หรือใช้ชื่อศูนย์ในการอ้างอิง เพื่อเผยแพร่สู่สาธารณะ ต้องได้รับอนุญาตเป็นลายลักษณ์อักษรจากศูนย์ฯ ก่อนทุกครั้ง
3. ทางห้องปฏิบัติการจะไม่รับผิดชอบต่อความเสียหายใดๆที่เกิดขึ้น ไม่ว่าโดยทางตรงหรือทางอ้อม ในกรณีที่นำข้อมูล ผลการทดสอบ หรือข้อสรุปในเอกสารฉบับนี้ไปใช้ในการออกแบบ การผลิต และเพื่อจุดประสงค์ใดก็ตาม
4. หากมีข้อสงสัยเกี่ยวกับรายงานผลการทดสอบ กรุณาติดต่อกลับภายใน 7 วันทำการ นับตั้งแต่วันที่ได้รายงานผลการทดสอบ
5. ห้องปฏิบัติการจะไม่รับผิดชอบข้อมูลจากลูกค้า กรณีที่ข้อมูลมีผลต่อการทดสอบ

Center of Plant-produced Biopharmaceutical for Dentistry

ศูนย์พัฒนากระบวนการผลิตชีวเภสัชภัณฑ์จากพืชเพื่อประยุกต์ใช้ทางทันตกรรม จุฬาลงกรณ์มหาวิทยาลัย
คณะทันตแพทยศาสตร์ จุฬาลงกรณ์มหาวิทยาลัย
34 ถ.อังรีดูนัง แขวงปทุมวัน เขตปทุมวัน กรุงเทพฯ

ผลวิเคราะห์ (Test Result)

เลขที่รายงาน: R65/056
ตัวอย่างที่ทดสอบ: ดังที่แสดงในตาราง
ลักษณะตัวอย่าง: ดังที่แสดงในตาราง

Sample code	Sample name	Sample description*	Image
65-Py-Pr-511	DeCsA110665.1	สารสกัดหลัง decarboxylation จากดอก CA2 Lot No.: FHC742	
65-Py-Pr-542	DeCsA110665.1W	สารสกัดหลัง decarboxylation ได้จากการล้างระบบสกัด หลังสกัดดอก CA2 และ CA1 Lot No.: FHC743	
65-Py-Pr-543	DeCsA110665.2	สารสกัดหลัง decarboxylation จากดอก CA1 Lot No.: FHC742	CS extract

Remark:

*ระบุข้อมูล Lot No, Mfg date, Exp date, Manufacturer (ถ้ามี)

Center of Plant-produced Biopharmaceutical for Dentistry

ศูนย์พัฒนากระบวนการผลิตชีวเภสัชภัณฑ์จากพืชเพื่อประยุกต์ใช้ทางทันตกรรม จุฬาลงกรณ์มหาวิทยาลัย

คณะทันตแพทยศาสตร์ จุฬาลงกรณ์มหาวิทยาลัย

34 ถ.อังรีดูนัง แขวงปทุมวัน เขตปทุมวัน กรุงเทพฯ

ผลวิเคราะห์ (Test Result)

เลขที่รายงาน: R65/056

☑ Qualitative analysis

Sample code	Sample name	Test Result	Remark
65-Py-Pr-511	DeCsA110665.1	☑ CBD ☐ CBC ☐ CBG ☐ CBN ☐ CBGA<br>☑ d9-THC ☐ CBDA ☐ THCA ☐ THCV	
65-Py-Pr-542	DeCsA110665.1W	☑ CBD ☐ CBC ☐ CBG ☐ CBN ☐ CBGA<br>☑ d9-THC ☐ CBDA ☐ THCA ☐ THCV	
65-Py-Pr-543	DeCsA110665.2	☑ CBD ☐ CBC ☐ CBG ☐ CBN ☐ CBGA<br>☑ d9-THC ☐ CBDA ☐ THCA ☐ THCV	CS extract

*☐ checked if present

Center of Plant-produced Biopharmaceutical for Dentistry

ศูนย์พัฒนากระบวนการผลิตชีวเภสัชภัณฑ์จากพืชเพื่อประยุกต์ใช้ทางทันตกรรม จุฬาลงกรณ์มหาวิทยาลัย
คณะทันตแพทยศาสตร์ จุฬาลงกรณ์มหาวิทยาลัย
34 ถ.อังรีดูนัง แขวงปทุมวัน เขตปทุมวัน กรุงเทพฯ

ผลวิเคราะห์ (Test Result)

เลขที่รายงาน: R65/056

☑ Quantitative analysis

		Unit %w/w						
Sample name	น้ำหนักสารสกัด (g)	CBD	CBDA	CBDX	THC	THCA	THCX	Remark
DeCsA110665.1	70.92	35.58	N.D.	35.58	0.86	N.D.	0.86	
DeCsA110665.1W	55.19	31.13	N.D.	31.13	1.09	N.D.	1.09	
DeCsA110665.2	112.47	57.20	N.D.	57.20	1.68	N.D.	1.68	CS extract

All samples were run in triplicate and SD was not shown.

Remark:

1. Cannabidiol (CBD), cannabinol (CBN), and dronabinol (Δ9-tetrahydrocannabinol; THC) are purchased from THC Pharm GmbH (Frankfurt, Germany). Cannabidiolic acid (CBDA), cannabigerol (CBG), cannabigerolic acid (CBGA), Δ9-tetrahydrocannabinolic acid (THCA), olivetolic acid (OAL) are purchased from Cayman chemical company (USA). Olivetol (OL) is purchased from Sigma-Aldrich Chemie GmbH (USA).

2. CBDX: CBD + (CBDA*0.877). THCX: THC + (THCA*0.877)

3. ND: Not detected. N/A: Not applicable

CU-BoD
Center of Plant-produced Biopharmaceutical for Dentistry

ศูนย์พัฒนากระบวนการผลิตชีวเภสัชภัณฑ์จากพืชเพื่อประยุกต์ใช้ทางทันตกรรม จุฬาลงกรณ์มหาวิทยาลัย
คณะทันตแพทยศาสตร์ จุฬาลงกรณ์มหาวิทยาลัย
34 ถ.อังรีดูนัง แขวงปทุมวัน เขตปทุมวัน กรุงเทพฯ

ผลวิเคราะห์ (Test Result)

เลขที่รายงาน: R65/056

☑ HPLC chromatogram result (if applicable)

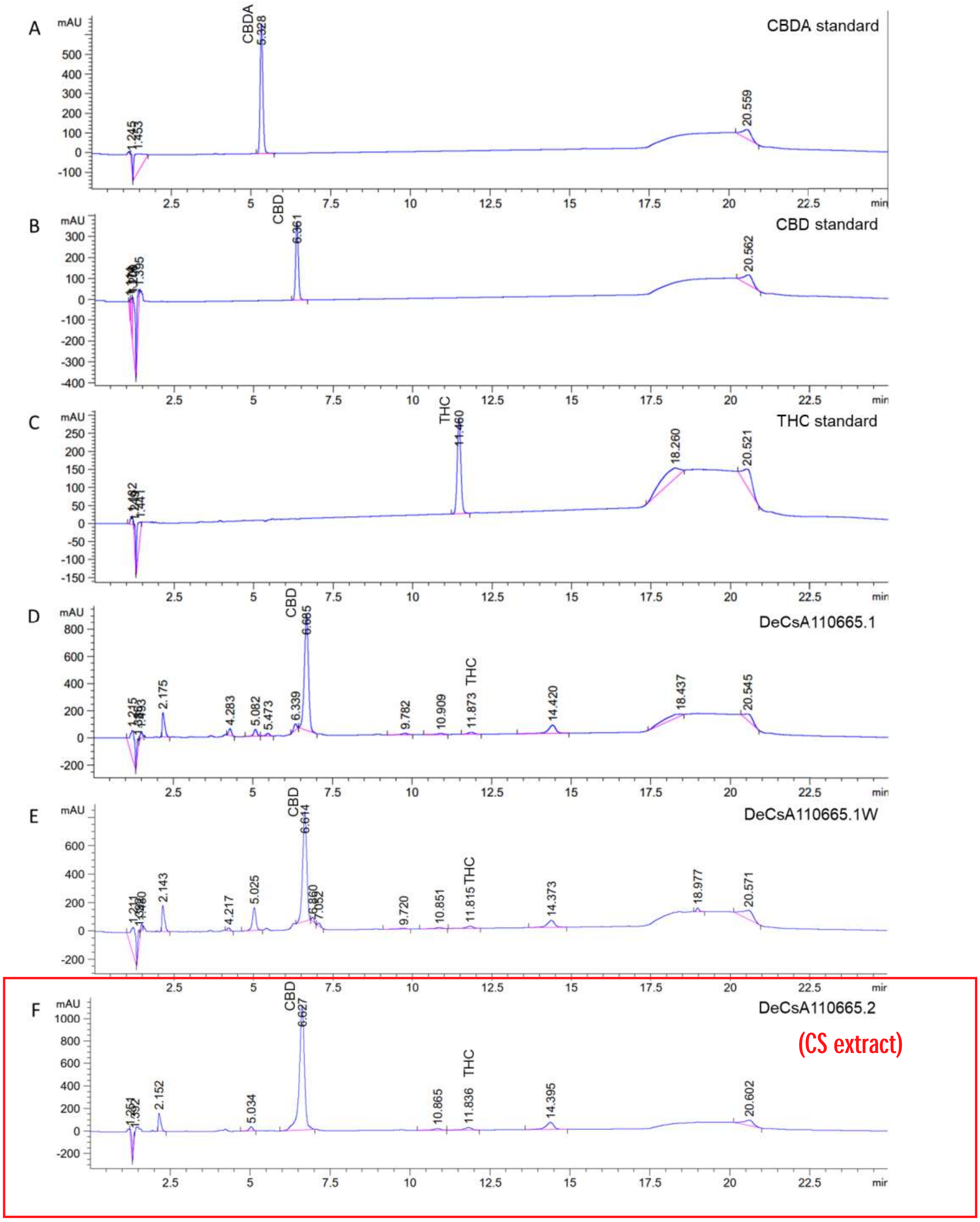

A: CBDA standard B: CBD standard C: THC standard

D: DeCsA110665.1 E: DeCsA110665.1W F: DeCsA110665.2

CU-BoD

Center of Plant-produced Biopharmaceutical for Dentistry

ศูนย์พัฒนากระบวนการผลิตชีวเภสัชภัณฑ์จากพืชเพื่อประยุกต์ใช้ทางทันตกรรม จุฬาลงกรณ์มหาวิทยาลัย

คณะทันตแพทยศาสตร์ จุฬาลงกรณ์มหาวิทยาลัย

34 ถ.อังรีดูนังต์ แขวงปทุมวัน เขตปทุมวัน กรุงเทพฯ

<u>ผลวิเคราะห์ (Test Result)</u>

เลขที่รายงาน: R65/056

สิ้นสุดรายงาน

ผู้ทดสอบ:

(ลงนาม) เพชรลดา โฆษิตานนท์ นักวิเคราะห์

(นางสาวเพชรลดา โฆษิตานนท์)
