# Supplementary material for: Enhanced Antibacterial and Anti-Inflammatory Activities of the Combination of Cannabis sativa and Propolis Extracts: An In Vitro Study
Source: Int J Mol Sci. 2025 Nov 19;26(22):11181. doi: 10.3390/ijms262211181 (PMC12652737; doi:10.3390/ijms262211181)
Supplement: Supplementary file 1 [file ijms-26-11181-s001.zip › Supplementary File S2 (Figures S1–S3).pdf]

Supplementary File S2

# Example of Combination 1 (Ratio 1:1) Preparation

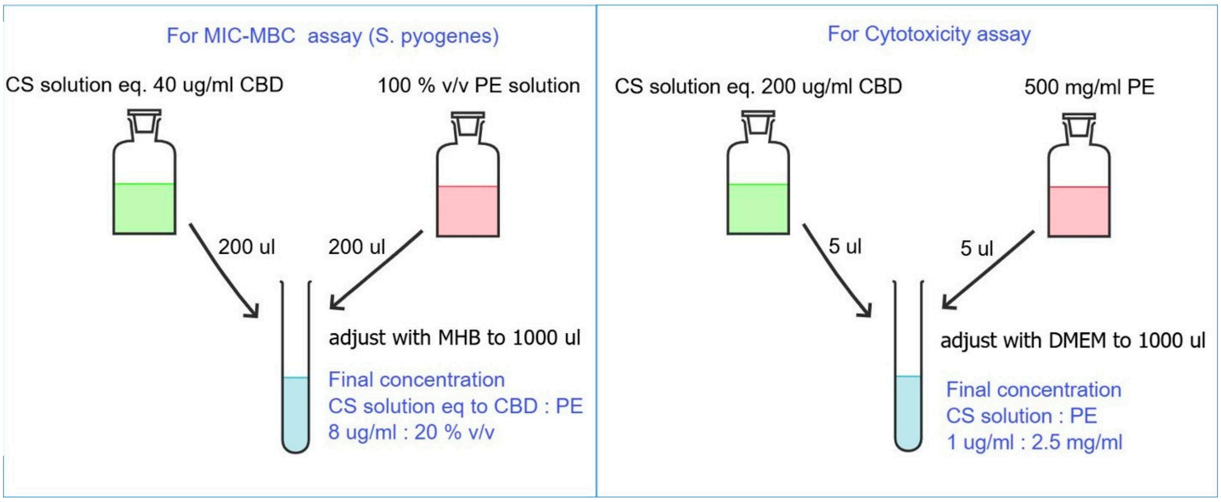


**Figure S1.** Preparation steps of Combination 1 (1:1 ratio) for biological evaluations.

**(Left)** For antibacterial evaluation using the MIC-MBC assay against *Streptococcus pyogenes*, 200 µL of $CS^{sol}$ (equivalent to 40 µg/mL CBD) was mixed with 200 µL of 100% v/v $PE^{sol}$. The mixture was then adjusted with MHB to a final volume of 1,000 µL. The final concentration of the combination was $CS^{sol}$ (CBD equivalent): $PE^{sol}$ = 8 µg/mL : 20% v/v.

**(Right)** For cytotoxicity and anti-inflammatory assays, 5 µL of $CS^{sol}$ (equivalent to 200 µg/mL CBD) and 5 µL of PE (500 mg/mL) were mixed and diluted with DMEM to a final volume of 1,000 µL. The final concentration of the combination was $CS^{sol}$:PE = 1 µg/mL : 2.5 mg/mL.

Supplementary File S2

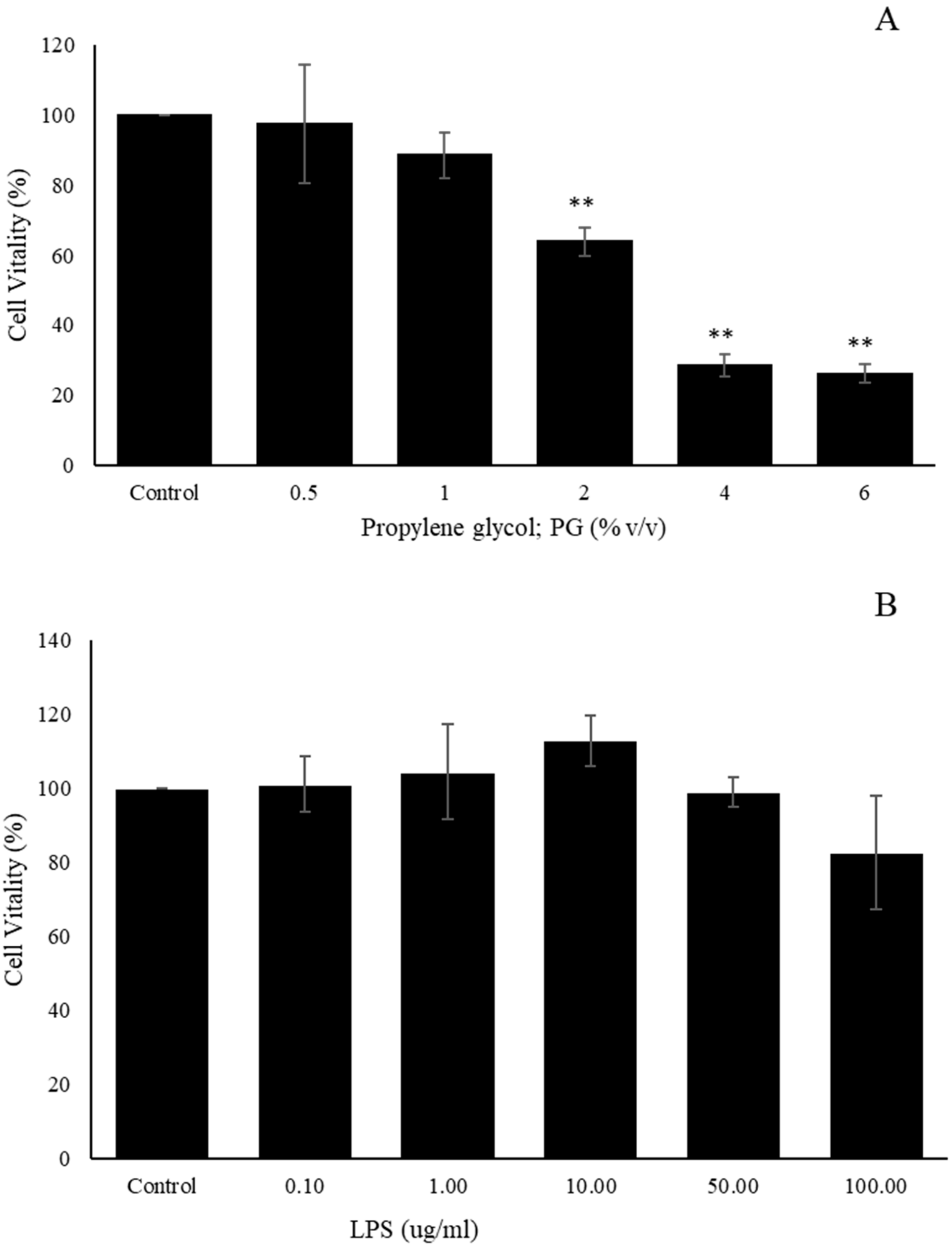


**Figure S2.** Viability of RAW264.7 cells after 24-hour treatment with (A) propylene glycol (PG), which was used as the solvent for $CS^{sol}$, and (B) lipopolysaccharide (LPS), which was used to induce inflammation. Cell viability was assessed using the MTT assay (n = 3). Results are expressed as mean ± standard error of the mean (SEM) and were analyzed using one-way ANOVA. *$p < 0.05$ and **$p < 0.01$ compared to the untreated control group.

Supplementary File S2

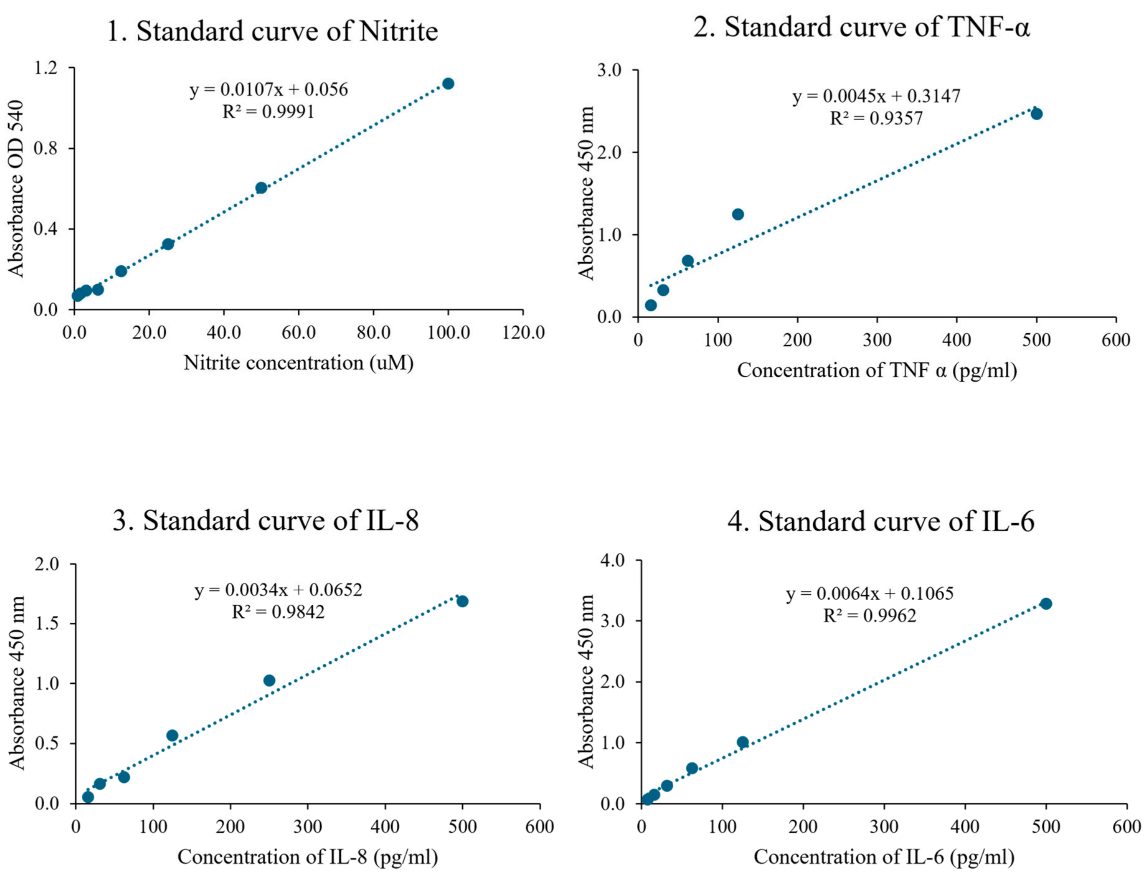


**Figure S3.** The standard curve of nitrate (1) with $R^2$ was used for the calculation of NO production, while the standard curves for TNF-α (2), IL-8 (3), and IL-6 (4), with their respective $R^2$ values, were used for the calculation of the cytokines TNF-α, IL-8, and IL-6, respectively.
